# Response to “Variable directionality of gene expression changes across generations does not constitute negative evidence of epigenetic inheritance” Sharma, A. Environmental Epigenetics, 2015, 1-5

**DOI:** 10.1186/s13059-016-0978-0

**Published:** 2016-05-16

**Authors:** Piroska E. Szabó

**Affiliations:** Van Andel Research Institute, Center for Epigenetics, 333 Bostwick Ave, Grand Rapids, MI 49503 USA

## Abstract

Abhay Sharma brings two arguments in favor of transgenerational epigenetic inheritance (TGEI) in mammals when criticizing our work. He uses probability calculations and finds that the probability of obtaining the number of common changes in the in utero-exposed prospermatogonia and the same cells in the next generation is significant in our study. He also compares our results to other published datasets and concludes that the probability for the observed overlap between independent studies is significant. We disagree with both arguments of Sharma and show here that his meta-analysis and statistical calculations are not correct.

Sharma [1] directly criticized our paper [2] in the first issue of *Environmental Epigenetics* in an attempt to defend the prevailing dogma of transgenerational epigenetic inheritance (TGEI) of environmentally induced changes in mammals [3, 4]. The journal (Michael Skinner, Editor in Chief) has not given us a chance to respond.

In the paper under dispute [2], we failed to find evidence supporting TGEI at the level of transcription and DNA methylation. We hypothesized that if an epigenetic aberration was triggered in the male fetal germ cells by environmental insult, it could be inherited by the sperm and might prevail in the fetal germ cells of the next, unexposed generation if not corrected by epigenome reprogramming. Part of our work involved assessing gene transcription in the fetal germ cells of the fetuses exposed in utero to endocrine disruptors and in fetal germ cells of the next generation (Table 4 in [2]). Any change would be considered inherited if it was affecting gene transcription similarly in the subsequent generations. Using rigorous statistics (1.5-fold change and FDR *P* <0.05) to interpret our RNA microarray results, we found an extremely low number of significant changes in the exposed germ cells (seven and two probes total for di(2-ethylhexyl)phthalate [DEHP] and vinclozolin [VZ] exposures, respectively) but none of these prevailed between generations. When we relaxed our statistical stringency (1.5-fold change and unadjusted *P* <0.05), we found one and eight probe overlaps between generations for DEHP and VZ, respectively. Every one of these changes occurred in the opposite direction in the subsequent generation. These findings using generally acceptable statistical rigor are consistent with the explanation that germline epigenetic reprogramming corrects the environment-induced mistakes in gene regulation.

In his first argument, Sharma selectively interpreted our results by focusing only on the next part of our analysis, when we further reduced the statistical stringency (1.05-fold change, *P* <0.05). He calculated the hypergeometric probabilities for the common hits between generations and reported them to reach statistical significance. At that extremely low level of statistical stringency we counted 284 and 325 genes that exhibited changes in subsequent generations for DEHP and VZ, respectively. Among these, only 77/325 and 30/284 occurred in the same direction (upregulated in two generations or downregulated in two generations). We suggest that the differences that are found in the opposite direction [2] may indicate a slight overcompensation in the erasure process. We disagree with Sharma’s opinion that a change that occurs in the opposite direction in subsequent generations constitutes an inherited epigenetic aberration. Further, a decrease of a gene transcript in one generation and an increase of the same transcript in the next generation cannot account for the same phenotype in the subsequent generations. Therefore, molecular changes occurring in the opposite direction in subsequent generations should not be combined in support of TGEI. Importantly, when we calculated the hypergeometric distribution probabilities of the number of changes that occurred in the same direction, those numbers were not significant: P(x > = 77) = 1 for DEHP and P(x > = 30) = 1 for VZ (Table 1).

**Table 1:** Hypergeometric probability calculations for the common changes defined using extremely low stringency (1.05-fold, *P* <0.05) detected in [2]

Number of genes (1.0 ST)	DEHP G1R (Iqbal)	DEHP G2R (Iqbal)	DEHP G1R-G2R (Iqbal)	Probability
21041	3639	1482	77 (same direction)	1
21041	3639	1482	248 (opposite direction)	0.73
21041	3639	1482	325 (both directions)	1.16E-06
Number of genes (1.0 ST)	VZ G1R (Iqbal)	VZ G2R (Iqbal)	VZ G1R-G2R (Iqbal)	Probability
21041	4295	1044	30 (same direction)	1
21041	4295	1044	254 (opposite direction)	8.94E-04
21041	4295	1044	284 (both directions)	4.04E-08

https://www.geneprof.org/GeneProf/tools/hypergeometric.jsp

Sharma argues [1]: “expression change in the opposite direction across generations does not necessarily compromise the positive evidence of transgenerational effects because phenotypic variability across generations is known in epigenetic inheritance. For example, transgenerational weakening and strengthening of phenotypes have been reported in several studies in mammals.” He also writes: “Moreover, gene expression changes in opposite direction across generations have also been reported in studies pertaining to effects of environmental agents in animals.” We would like to point out that the papers cited by Sharma report results regarding: (1) a single generation; (2) intergenerational changes that occur in the same direction; (3) transgenerational phenotypic (not molecular) changes that occur in the same direction between three generations; (4) are non-mammalian examples; or (5) concern miRNA. Specifically, none of these cited papers reports a TGEI phenotype that can be explained by a molecular change in the opposite direction between the generation exposed as germ cells and the next, unexposed, generation (or in the exposed germ cell and germ cell of the next generation). Brieno-Enriquez [5] reports that the embryonic phenotype, primordial germ cell (PGC) number defect at 13.5 dpc and apoptosis at 13.5 dpc, occur in F1 and F2 PGCs but not in F3 PGCs. The Let-7/LIN28/BLIMP1 pathway is misregulated in F1, F2, and F3 PGCs. The direction of change is the same for each component between F1 and F2 PGCs, exactly when the phenotypic changes that can be anticipated from misregulated BLIMP1 are actually observed in embryonic germ cells. The direction of change is opposite for certain components in the F3 PGCs, when the phenotype is no longer observed.

For his second argument [1], Sharma compares our data [2] to previously published rat and mouse datasets using hypergeometric probability calculations. He writes [1]: “In this analysis of mouse and rat gene expression data from diverse studies, the entire set of human genes listed in the Gene ontology was used for normalization, to find overlap between previous gene sets and genes identified by Iqbal and colleagues. The premise of this analysis was that a higher-fold enrichment with greater statistical significance in VZ-VZ comparison relative to VZ-DEPH comparison would indicate TGEI. Importantly, a higher enrichment with greater significance is observed for VZ-VZ overlap than VZ-DEPH overlap.” To the contrary of his expectations, a lack of probability of inter-study overlap is apparent in Figure 2 of Sharma [1], where significance values should accompany the columns according to the figure legend as they do in [6], but only the columns pertaining to all 36 datasets combined are labeled with statistical value. Using the same gene lists Sharma used [1] (and one should use with caution, see below), we also counted the common hits and calculated the hypergeometric probabilities of finding common hits between our study [2] and previous studies (Supplement 2 in [1], References to source data therein). As displayed in Table 2, two comparisons are marginally significant but the probability for finding an overlap pertaining to all 36 datasets combined is not statistically significant. We note that probabilities were calculated without considering adjustments for multiple testing. If we accounted for that, these probabilities would be even larger.

**Table 2:** Hypergeometric probability calculations for common VZ-induced changes between our study [2] and previous work considered by Sharma [1]

Previous dataset	Number of differentially expressed genes in dataset	Common with VZ G1R-G2R (Iqbal)	Probability	Common with DEHP G1R-G2R (Iqbal)	Probability
		*P* = 21041		*P* = 21041	
		p(VZ) = 284		p(DEPH) = 325	
F1 embryo male testis	83	0	1	0	1
F1 embryo E13 male testis	309	5	0.40	3	0.86
F1 embryo E14 male testis	162	2	0.65	2	0.72
F1 embryo E16 male testis	135	3	0.27	1	0.88
F2 embryo male testis	83	0	1	0	1
F3 embryo male testis	83	0	1	0	1
F3 adult male sertoli cells	383	10	0.04	9	0.14
F3 adult female granulosa cells	419	7	0.34	7	0.47
F3 adult female heart	113	0	1	2	0.52
F3 adult female kidney	451	9	0.16	9	0.26
F3 adult female liver	221	0	1	1	0.97
F3 adult female uterus	230	5	0.20	5	0.28
F3 adult male heart	113	1	0.79	2	0.52
F3 adult male kidney	93	2	0.36	1	0.77
F3 adult male liver	55	1	0.53	1	0.58
F3 adult male prostate	825	12	0.44	9	0.89
F3 adult male seminal vesicle	180	2	0.70	0	1
F3 adult female ovary	2555	29	0.86	31	0.94
F3 adult male testis	338	3	0.84	2	0.97
F3 adult male amygdala	224	2	0.81	1	0.97
F3 adult male hippocampus	71	1	0.62	0	1
F3 adult female amygdala	103	0	1	0	1
F3 adult female hippocampus	910	12	0.57	18	0.17
F3 adult male brain	642	3	0.99	5	0.97
F3 adult male basolateral amygdala	40	0	1	0	1
F3 adult male brain cortex	57	2	0.18	1	0.59
F3 adult male hippocampus CA1	74	0	1	0	1
F3 adult male hippocampus CA3	212	2	0.03	1	0.96
F3 adult female basolateral amygdala	48	1	0.48	0	1
F3 adult female brain cortex	79	0	1	0	1
F3 adult female hippocampus CA1	35	1	0.38	1	0.38
F3 adult female hippocampus CA3	25	0	1	0	1
F3 adult male ventral prostate	685	5	0.96	8	0.83
F3 adult male prostate epithelial cells	215	2	0.79	0	1
F3 embryo E13 male primordial germ cells	404	3	0.91	1	0.99
F3 embryo E16 male prospermatogonia	114	0	1	0	1
Cumulative (36 studies)	10769	125	0.96	121	0.99

There are several problems with Sharma’s interpretations [1]: (1) he did not acknowledge the lack of common hits at the rigorous statistical cutoff value but only focused on the findings using extremely low statistical stringency; (2) he made the assumption that the changes occurring in the opposite direction between studies could be considered common changes; (3) he calculated the probability of obtaining the exact number of successes in the sample (not the probability of obtaining at least that many successes); (4) he did not apply the same statistical cutoff values between studies; (5) he used the number of known human genes as reference, but this analysis should be limited to what can be detected by the specific array; (6) he considered studies that did not actually search for transgenerational changes in germ cells; and (7) none of his study-to study comparisons reached significant probability values in his Figure 2, yet he concluded finding more significant overlap for VZ-VZ than VZ-DEHP comparisons.

Based on the results of our study [2] and our reassessment of Sharma’s calculations we cannot provide evidence in support of TGEI in mammals.
